# Supplementary material for: Mechanisms governing the pioneering and redistribution capabilities of the non-classical pioneer PU.1
Source: Nat Commun. 2020 Jan 21;11:402. doi: 10.1038/s41467-019-13960-2 (PMC6972792; doi:10.1038/s41467-019-13960-2)
Supplement: Supplementary file 7 — Source data [file 41467_2019_13960_MOESM7_ESM.zip › Source_Data/FigureS2/FigureS2B_MotifScanOutput/KmeansCluster3/homerResults/motif7.similar.html]

motif7

## Information for motif7

C
G
A
T
C
A
T
G
A
G
T
C
G
A
C
T
G
A
C
T
T
A
G
C
G
A
T
C
G
A
C
T
A
T
G
C
G
A
C
T
A
G
C
T
A
G
T
C
  
Reverse Opposite:  

T
C
A
G
T
C
G
A
C
T
G
A
T
A
C
G
C
T
G
A
C
T
A
G
A
T
C
G
C
T
G
A
C
T
G
A
T
C
A
G
G
T
A
C
G
C
T
A
  

|  |  |
| --- | --- |
| p-value: | 1e-150 |
| log p-value: | -3.476e+02 |
| Information Content per bp: | 1.606 |
| Number of Target Sequences with motif | 1090.0 |
| Percentage of Target Sequences with motif | 22.86% |
| Number of Background Sequences with motif | 3881.9 |
| Percentage of Background Sequences with motif | 9.15% |
| Average Position of motif in Targets | 104.8 +/- 49.0bp |
| Average Position of motif in Background | 104.5 +/- 90.2bp |
| Strand Bias (log2 ratio + to - strand density) | -0.1 |
| Multiplicity (# of sites on avg that occur together) | 1.23 |
| Motif File: | file (matrix) reverse opposite |

### Similar de novo motifs found

|  |  |  |  |  |  |  |  |
| --- | --- | --- | --- | --- | --- | --- | --- |
| Rank | Match Score | Redundant Motif | P-value | log P-value | % of Targets | % of Background | Motif file |
| 1 | 0.752 | C T A G T C G A C T A G C T A G C T G A T C G A A T C G C T G A | 1e-105 | -242.156088 | 36.61% | 21.84% | motif file (matrix) |
| 2 | 0.764 | A T G C G C A T A C T G T A G C A G C T A G C T T A G C A G C T G C A T G T A C G C A T | 1e-76 | -175.679171 | 19.98% | 10.29% | motif file (matrix) |
| 3 | 0.784 | A G C T A G T C G A T C A G C T A T G C G A T C G A C T G A T C G A T C G A C T A G T C | 1e-72 | -167.269800 | 5.81% | 1.35% | motif file (matrix) |
| 4 | 0.777 | A C T G T C G A C T G A T A C G T C G A C G T A T A C G C G T A C G T A T A C G T G C A C T G A T A C G T C G A | 1e-13 | -32.086538 | 0.96% | 0.20% | motif file (matrix) |
